# FDA expanded approval of Pluvicto (Lutetium-177 vipivotide tetraxetan) in metastatic castration-sensitive prostate cancer

**DOI:** 10.1097/MS9.0000000000004548

**Published:** 2025-12-09

**Authors:** Malakia Saqib, Bilal Arshad, Sakan Binte Imran, Ehtisham Haider

**Affiliations:** a Department of Medicine, Khyber Medical College, Peshawar, Pakistan; b Department of Medicine, Shifa College of Medicine, Islamabad, Pakistan; c Department of Medicine, Sir Salimullah Medical College, Dhaka, Bangladesh; d Department of Medicine, Punjab Medical College, Faisalabad, Pakistan

*To the Editor,*

With a narrow range of therapeutic approaches, prostate cancer, characterized by the uncontrolled growth of prostate cells in men, ranks as the second most common cause of cancer-related deaths. Usually, an invasive biopsy is used as a confirmation of a prostate-specific antigen (PSA) test and digital rectal examination; the disease itself can develop anywhere in the gland. For the disease occurrence, the prostate cells undergo malignant transformation, which then progresses from prostatic intraepithelial neoplasia to prostate adenocarcinoma and eventually metastasis, most commonly to bones, followed by lymph nodes, lungs, and brain^[1]^. The usual first-line treatment for hormone-sensitive prostate cancer (HSPC) is still androgen deprivation therapy (ADT); nevertheless, resistance frequently results in castration-resistant illness^[2]^. It received its first approval on 23 March 2022, for metastatic castration-resistant prostate cancer, targeted radioligand therapy with Pluvicto (Lutetium-177 vipivotide tetraxetan) has now been approved for metastatic castration-sensitive prostate cancer (mCSPC), showing encouraging results due to its precise tumor targeting and reduced systemic toxicity. This manuscript complies with TITAN Guidelines, 2025, declaring no use of AI^[3]^.

Traditionally, androgen deprivation therapy (ADT) combined with androgen receptor pathway inhibitors (ARPIs), irrespective of taxane chemotherapy, has been the mainstay of management and treatment for metastatic prostate cancer. The β-emitting radiopharmaceutical^177^Lu-vipivotide tetraxetan ([^177^Lu]Lu-PSMA-617), marketed as Pluvicto, which specifically targets PSMA, received approval for PSMA-expressing metastatic castration-resistant prostate cancer (mCRPC) after treatment failure with ADT and ARPIs. Situations warranting a delay in taxane therapy allowed administration prior to chemotherapy^[4]^. In the pivotal VISION study, adding Pluvicto to standard care significantly improved overall survival and radiographic progression-free survival (rPFS) compared to standard therapy alone. Similarly, in the phase 2 TheraP trial conducted in the post-docetaxel mCRPC setting, Pluvicto demonstrated superior intermediate outcomes, such as PSA response, compared to cabazitaxel^[5]^. Since PSMA is minimally expressed in normal tissues but highly upregulated in prostate cancer cells, the radioligand can deliver targeted β-particle radiation directly to tumors while sparing healthy organs, thereby reducing systemic toxicity.

More recently, the radioligand “[177Lu]Lu-PSMA-617” was approved for use in mCSPC, extending its clinical indications. Only a few cases of its use prior to ADT in metastatic prostate cancer have been documented. Two cycles of [177Lu]Lu-PSMA-617 were administered to a patient who had metastatic recurrence following radical prostatectomy and adjuvant radiotherapy; the patient experienced a full radiographic response. A total of 10 patients with low-volume mHSPC were treated with two cycles of [177Lu]Lu-PSMA-617 without concurrent ADT in a pilot study by Privé *et al*. Half of the patients demonstrated a PSA drop of more than 50%, and one patient experienced complete radiographic remission. In a case report by Giri *et al*^[4]^, a patient with multifocal mHSPC and no prior local or systemic therapy received two cycles of [177Lu]Lu-PSMA-617 as primary therapy, resulting in a 95% PSA reduction, improvement of urinary symptoms, and near-complete resolution of primary, nodal, and osseous lesions. These findings demonstrate the prospects of [177Lu]Lu-PSMA-617 in the context of frontline treatment and draw attention to the need for prospective trials to evaluate its long-term efficacy and safety^[4]^. Another recent study reported that ^177^Lu-PSMA-617, followed by docetaxel, improved antitumor activity in patients with de novo high-volume metastatic HSPC compared with docetaxel alone, without increased toxic effects, further supporting its potential role in earlier stages of the disease^[6]^.

Taken together, Pluvicto’s (^177^Lu-PSMA-617) recent FDA approval for treatment in mCSPC marks a major breakthrough in personalized oncology. This radioligand therapy provides great efficacy with low toxicity by precisely targeting PSMA, enabling individualized treatment and possibly postponing the need for chemotherapy. It reflects promising efficacy and safety in both castration-resistant and hormone-sensitive metastatic prostate cancer. Evidence that is coming to light from pilot studies and case reports highlights its potential as an earlier-line therapy, either alone or in conjunction with existing treatment modalities. Prospective clinical trials are warranted to establish its long-term benefits, optimal sequencing, and role in the frontline management of metastatic prostate cancer.

## Data Availability

Not applicable.
